# 经典瞬时受体电位通道蛋白在人非小细胞肺癌组织中的表达

**DOI:** 10.3779/j.issn.1009-3419.2010.06.009

**Published:** 2010-06-20

**Authors:** 奇 张, 建行 何, 文菊 卢, 伟强 殷, 海虹 杨, 小明 徐, 道远 汪

**Affiliations:** 510120 广州，广州医学院第一附属医院呼吸疾病国家重点实验室，广州医学院第一附属医院胸外科 State Key Lab of Respiratory Diseases, the first affiliated hospital of Guangzhou Medical College, Guangzhou Medical College, Guangzhou 510120, China; Department of Thoracic Surgery, the First Affiliated Hospital of Guangzhou Medical College, Guangzhou 510120, China

**Keywords:** 肺肿瘤, 经典瞬时受体电位通道蛋白, 钙池操纵性钙内流, Lung neoplasms, Transient receptor potential canonical proteins, Store-operated calcium entry

## Abstract

**背景与目的:**

经典瞬时受体电位（transient receptor potential canonical, TRPC）通道蛋白是一种非选择性阳离子通道蛋白家族，主要位于细胞膜表面，对钙离子具有通透性。研究认为，TRPC可能构成钙池操纵性钙通道（store-operated calcium channels, SOCC）并介导钙池操纵性钙内流（store-operated calcium entry, SOCE），从而参与细胞的增殖、迁移、基因转录等生命活动。本研究检测非小细胞肺癌（non-small cell lung cancer, NSCLC）组织中TRPC mRNA及蛋白质的表达情况，初步探讨TRPC与NSCLC的可能关系。

**方法:**

建立TRPC1-7等7个家族成员的荧光定量PCR检测方法，对24例NSCLC患者的肿瘤组织进行了TRPC mRNA的定量检测，并通过蛋白质免疫印迹法对TRPC在蛋白质水平的表达进行了验证。

**结果:**

在NSCLC患者癌组织检测到TRPC1、TRPC3、TRPC4和TRPC6 mRNA的表达，未检测到TRPC2、TRPC5和TRPC7 mRNA的表达。肺癌组织中TRPC表达丰度为：TRPC1≈TRPC6>TRPC3>TRPC4。蛋白质免疫印迹证实了非小细胞肺癌组织中TRPC1、TRPC3、TRPC4和TRPC6在蛋白质水平的表达。

**结论:**

非小细胞肺癌组织在mRNA和蛋白质水平均表达TRPC1、TRPC3、TRPC4和TRPC6，其中主要表达TRPC1和TRPC6，它们在构成肺癌细胞中SOCC、介导产生SOCE中的作用有待进一步研究。

## Abstract (en)

**Background and objective:**

Transient receptor potential canonical (TRPC) proteins, a group of Ca^2+^ permeable nonselective cation channels, are thought to constitute store-operated calcium channels (SOCC) and mediate store-operated calcium entry (SOCE) in various cell types. Members of TRPC have been found to be involved in abnormal proliferation, differentiation, and growth of cancer cells. The aim of this study is to detect the mRNA and protein expression of TRPC in non-small cell lung cancer (NSCLC).

**Methods:**

Real-time quantitative PCR was performed to screen the expression of TRPC mRNA in NSCLC tissue. Protein expression of TRPC was detected by Western blot.

**Results:**

Among the seven family members of TRPC so far identified (TRPC1-7), we detected the expression of TRPC1, TRPC3, TRPC4, TRPC6 mRNA in 24 cases of NSCLC tissue; TRPC2, TRPC5 and TRPC7 mRNA were not detectable. The relative abundance of the expressed TRPC was TRPC1≈TRPC6>TRPC3>TRPC4. Western blot confirmed the protein expression of TRPC1, TRPC3, TRPC4 and TRPC6 in NSCLC tissue.

**Conclusion:**

Out of the seven members of TRPC, we found TRPC1, TRPC3, TRPC4, TRPC6 mRNA and protein were selectively expressed in human NSCLC tissue. This study could provide a basis for future exploration of the individual role of these TRPC proteins in mediating SOCE and in the progression of lung cancer.

钙离子（Ca^2+^）作为细胞内第二信使，广泛参与细胞各种生命活动。细胞内Ca^2+^浓度（[Ca^2+^]_i_）调节主要依靠：肌浆内质网（ER/SR）钙池系统中Ca^2+^释放以及胞外Ca^2+^通过离子通道如电压依赖性钙通道（voltage dependent calcium channels, VDCC）、钙池操纵性钙通道（storeoperated calcium channels, SOCC）或受体操纵性钙通道（receptor-operated calcium channels, ROCC）内流^[1]^。质膜系统钙离子泵和离子交换蛋白同时参与细胞内Ca^2+^的调节，从而平衡着胞浆与细胞器之间的钙水平。经典瞬时受体电位（transient receptor potential canonical, TRPC）通道蛋白为非选择性阳离子通道家族，包括7个成员，即TRPC1-7。研究表明，TRPC中的部分成员可能参与构成钙池操纵性钙通道SOCC，并介导产生钙池操纵性钙内流（Store-operated calcium entry，SOCE，过去称CCE）；TRPC介导的SOCE在细胞增殖、迁移和基因转录等细胞活动中发挥重要作用^[2]^。本研究应用实时荧光定量PCR（Real-time fluorescence quantitative PCR, RT-qPCR）和蛋白质免疫印迹技术首次检测了TRPC mRNA和蛋白质在非小细胞肺癌（non-small cell lung cancer, NSCLC）组织中的表达情况，并初步分析了TRPC与NSCLC细胞中SOCE的形成可能存在的关系，为进一步开展TRPC的功能研究奠定了分子基础。

## 1 材料与方法

### 1.1 材料

#### 1.1.1 肺癌组织标本的分离与贮存

标本来自于广州医学院第一附属医院呼吸疾病研究所胸外科于2007年10月-2009年3月间收集的24例病例。所有患者术前未做化疗、放疗及其它抗肿瘤治疗；其中男13例，女11例，中位年龄56.5岁（32岁-79岁）；术后病理确诊为NSCLC，病理类型包括：腺癌15例，鳞癌9例。根据1997年UICC分期包括：Ⅰa期5例，Ⅰb期3例，Ⅱa期2例，Ⅱb期2例，Ⅲa期8例，Ⅲb期2例，Ⅳ期2例。所有新鲜标本在液氮中冻存。

#### 1.1.2 主要仪器与试剂

iCycler IQ^5^荧光定量PCR仪以及微型凝胶垂直电泳装置购自Bio-Rad公司。Trizol试剂盒为Invitrogen公司（美国）产品。逆转录采用iScript逆转录试剂盒（Bio-Rad公司，美国）。荧光定量PCR试剂使用QuantiTect SYBR Green（Qiagen公司，美国）。引物由TAKARA公司合成。TRPC1、TRPC3、TRPC4和TRPC6和β-actin抗体分别购自Alomone公司（以色列）和Santa cruz公司（美国）。聚偏二氟乙烯膜（PVDF膜）、分子量标准Precision Plus和ECL发光液采用Bio-Rad公司产品。RIPA组织裂解液购自上海博彩生物公司。Tris、甘氨酸、Tween-20、DTT、BSA等购自Sigma公司（美国）。

### 1.2 方法

#### 1.2.1 总RNA抽提

应用Trizol试剂，按公司产品说明书提取总RNA。用TURBO DNA-*free*试剂（Ambion公司）除去总RNA样品中残留的DNA。紫外分光光度法测定*A*_260 nm_、*A*_280 nm_光密度，按公式[RNA]（μg/mL）= 40×稀释倍数×*A*_260 nm_计算RNA浓度；用于本研究的RNA样品质量要求是，*A*_260_与*A*_280_比值>1.8，甲醛变性琼脂糖凝胶电泳清晰可见28s和18s两条区带，带型无弥散。

#### 1.2.2 RNA逆转录

反应体系体积为20 μL，其中含RNA 1 μg，按iScript逆转录试剂盒的说明配制反应体系。逆转录条件为：25 ℃、5 min；42 ℃、30 min；85 ℃、5 min。产物在-80 ℃保存。

#### 1.2.3 荧光实时定量PCR

按QuantiTect SYBR Green PCR试剂盒要求配制反应体系：PCR反应总体系25 μL，其中含cDNA 1 μL。引物设计采用Primer 3软件（http://frodo.wi.mit.edu/cgi-bin/primer3/primer3_www.cgi），序列见表 1，其中Importin（IPO8）^[3]^为内参基因，用以排除样本间上样量的差异。各样本设置3个复孔，以无模板反应孔为阴性对照，人脑组织cDNA为阳性对照^[4]^。PCR扩增条件参照文献^[5]^：①预变性95 ℃、15 min；②94 ℃、15 s，57.5 ℃、20 s，72 ℃、20 s，共45个循环；③95 ℃、1 min；④55 ℃、1 min；⑤55 ℃→95 ℃ Δt0.5 ℃、10 s。反应产物行1.5%琼脂糖凝胶电泳，并送TAKARA公司测序，以验证扩增产物的长度和序列，以及扩增的特异性。采用阳性对照（人脑组织）CDNA，进行5个梯度的等比稀释，荧光定量PCR检测得到各基因引物的标准曲线和扩增效率。

**1:** 人TRPC引物信息表 Human TRPC primers' sequences and products length

*TRPC* gene	Gene Bank No.	Primer（left/right）	Length (bp)
*TRPC1*	NM_003304	5’-ttgtggaggtggaattcagg-3’	148
		5’-cgtttgtcaagaggctcgtc-3’	
*TRPC2*	NR_002720	5’-tcatggtcattgtgctgctc-3’	84
		5’-actccacgtcagcatcatcc-3’	
*TRPC3*	NM_003305	5’-cagccaacacgttatcagca-3’	172
		5’-cctcagttgcttggctcttg-3’	
*TRPC4*	NM_001135958	5’-cgaaagggttaacctgcaaa-3’	83
		5’-cagggactgcagtgtctcaa-3’	
*TRPC5*	NM_012471	5’-gtgctgctgaacatgctgat-3’	94
		5’-gcttcgtccttgcaaacttc-3’	
*TRPC6*	NM_004621	5’-cagacaatggcggtcaagtt-3’	117
		5’-tggtccacgcattatcttcc-3’	
*TRPC7*	NM_020389	5’-gttaaaaccctgccaaacga-3’	143
		5’-tcccagatttccttgcattc-3’	
*IPO8*	NM_006390	5’-aaccaaggggtggttcattc-3’	120
		5’-ttgccacagctcttcatcct-3’	

#### 1.2.4 蛋白质免疫印迹分析

① 组织总蛋白的提取：从-196 ℃冰箱取出非小细胞肺癌组织及正常肺组织，在冰上迅速研磨成粉末，按每100 mg组织加入1 mL RIPA组织裂解液，冰上孵育30 min；于4 ℃、12 000 g离心30 min，收集上清液，为提取的总蛋白；②组织总蛋白定量：采用Bradford方法检测蛋白浓度；③10%SDS-聚丙烯酰胺凝胶电泳：蛋白质于100 ℃加热5 min进行热变性，上样量为30 μg；④免疫印迹：采用湿法转膜、5%脱脂奶粉4 ℃封闭过夜。TRPC一抗进行1:1 000稀释后孵育膜2 h，二抗按1:5 000稀释后孵育膜1 h。用ECL发光液对信号进行检测。X光片曝光、显影、定影。实验以β-actin为上样内参。

#### 1.2.5 数据分析及统计处理

根据各基因引物效率以及未知样品的Ct值，采用Pfaffl方法^[6]^，计算目的基因表达的初始拷贝数；以IPO8为内参，计算各样本*TRPC*基因与内参基因*IPO8*的比值，为基因的相对表达量。组间差异的统计学分析采用*t*检验。

## 2 结果

### 2.1 荧光定量PCR结果

本研究以Gene Bank中发表的mRNA序列为模板，分别设计合成了特异性针对人*TRPC1-7*基因和*IPO8*内参基因mRNA的荧光定量PCR扩增引物。以人脑组织cDNA为阳性对照制作荧光定量PCR标准曲线，经反复测定（>5次），筛选得到扩增效率接近100%、扩增产物的溶解曲线呈现单一峰的一组引物（表 1）。另外，扩增产物行琼脂糖凝胶电泳见单一条带且同预计分子量吻合（图 1），经测序证实与靶序列匹配率大于99%。

**1:**
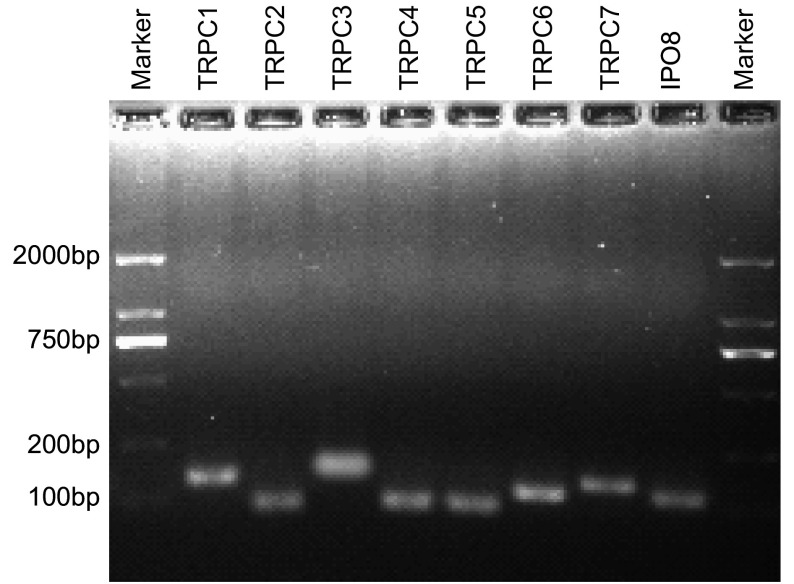
人脑组织（阳性对照）TRPC和IPO8荧光定量PCR扩增产物的琼脂糖凝胶电泳结果。采用所设计的引物（表 1）分别检测到TRPC1-7和IPO8在人脑组织中的表达。电泳显示各基因扩增产物条带单一、大小与预期分子量一致，表明引物特异性好 Agarose gel electrophoresis of TRPC and IPO8 qPCR products. Human brain cDNA was used as the positive control to validate the specificity of TRPC1-7 and IPO8 qPCR primers. Agarose gel electrophoresis indicated a single band with expected size of each qPCR product (TRPC1-7 and IPO8)

应用这些引物，对24例NSCLC患者癌组织中的TRPC进行了检测，结果发现（图 2）：NSCLC组织中高表达TRPC1和TRPC6，少量表达TRPC3和TRPC4，统计学分析显示各基因的相对表达丰度为TRPC1≈TRPC6>TRPC3>TRPC4，其中TRPC3和TRPC4的表达量分别约为TRPC1的1/8和1/25（两两均值之比，*P*=0.000 4）；在NSCLC组织中未检测到TRPC2、TRPC5和TRPC7 mRNA的表达。

**2:**
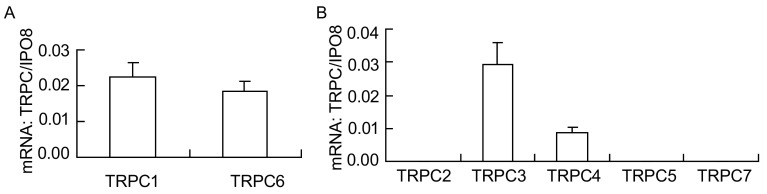
TRPC mRNA在NSCLC组织中的表达谱及表达丰度。荧光定量PCR检测到NSCLC组织表达TRPC1、TRPC3、TRPC4和TRPC6 mRNA，未检测到TRPC2、TRPC5或TRPC7 mRNA的表达；其中TRPC mRNA相对表达丰度为：TRPC1≈TRPC6>TRPC3>TRPC4。A：NSCLC组织高表达TRPC1和TRPC6；B：TRPC3和TRPC4在NSCLC组织中呈较低水平低表达，其表达量分别为TRPC1的1/8和1/25（*P*=0.000 4）；未检测到TRPC2、5或7的表达 Expression profile of TRPC mRNA in NSCLC tissue. TRPC1, TRPC3, TRPC4 and 6 mRNA were detected in NSCLC tissues by real-time qPCR. TRPC2, TRPC5 and TRPC7 mRNA were not detectable; the relative abundance of TRPC mRNA was TRPC1≈TRPC6 >TRPC3>TRPC4. A: TRPC1 and TRPC6 mRNA were highly expressed in NSCLC tissue; B: The expression of TRPC3 and TRPC4 in NSCLC was relatively low, which was about 1/8 and 1/25 comparing to TRPC1 (*P* =0.000 4); TRPC2, TRPC5 and TRPC7 mRNA were not detectable

### 2.2 TRPC蛋白质免疫印迹结果

以β-actin为内参蛋白，对NSCLC组织中mRNA水平有表达的*TRPC1*、*TRPC3*、*TRPC4*和*TRPC6*基因，应用免疫印迹方法验证了其蛋白质的表达，结果表明NSCLC组织表达TRPC1、TRPC3、TRPC4和TRPC6通道蛋白（图 3）。

**3:**
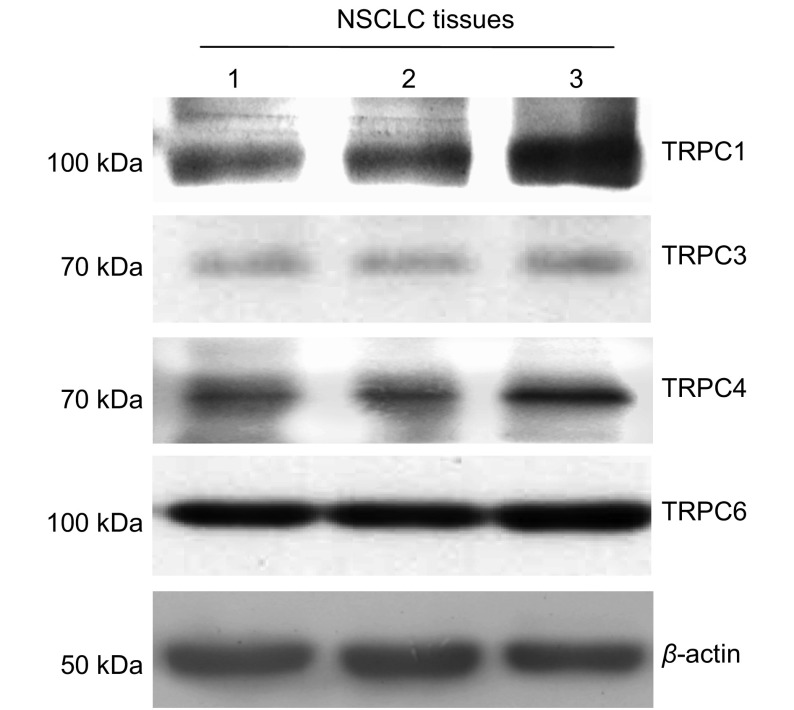
免疫印迹法检测NSCLC组织中TRPC1、TRPC3、TRPC4和TRPC6的表达。蛋白质免疫印迹显示TRPC1、TRPC3、TRPC4和TRPC6蛋白条带，*β*-actin为上样内参蛋白，免疫杂交信号强度均一，表明NSCLC组织中表达TRPC1、TRPC3、TRPC4和TRPC6通道蛋白。图中所示为取自3例不同NSCLC组织的代表性数据 Detection of TRPC1, TRPC 3, TRPC 4 and TRPC 6 protein expression in NSCLC tissue by Western blot. Representative data of Western blot from 3 different patients indicate TRPC1, TRPC 3, TRPC 4 and TRPC 6 proteins were expressed in NSCLC tissue. *β*-actin was used as loading control

## 3 讨论

细胞内Ca^2+^的稳态平衡与细胞维持正常的生命活动有密切的关系。在生理条件下，SOCC介导的SOCE是非兴奋细胞产生钙离子内流、维持细胞内Ca^2+^的稳态平衡的重要途径之一：细胞外激动剂作用于G蛋白偶联受体（G protein-coupled receptor, GPCR）产生三磷酸肌醇（inositol triphosphate, IP3），IP3与钙池上受体结合后受体开放并迅速释放Ca^2+^入胞浆，钙池出现钙损耗，效应蛋白感受钙池损耗状态并将信号传递给位于细胞膜的SOCC，后者活化开放引起细胞外Ca^2+^迅速内流形成SOCE^[7]^。目前，关于TRPC成员在构成SOCC、介导产生SOCE中的作用虽然仍有争议，但越来越多的研究^[2, 8]^发现，TRPC1、TRPC4和TRPC6在多种不同类型细胞均具有SOCC的功能，比如：在增生的人肺动脉平滑肌细胞，TRPC1表达增加、SOCE增强，经TRPC1反义寡核苷酸处理后，人肺动脉平滑肌细胞增殖和SOCE均受到抑制^[9]^；在*TRPC4*基因敲除小鼠血管内皮细胞上记录的SOCE要比TRPC4野生型小鼠低^[10]^；应用TRPC6反义寡核甘酸能够降低大鼠肺动脉平滑肌细胞表达TRPC6，血小板源性生长因子刺激的SOCE同时受到抑制^[11]^；TRPC3在低表达时可能参与构成SOCC，但大多数研究^[12]^认为TRPC3不具有SOCC的功能。病理条件下，SOCE可能发生变化并参与疾病的病理机制^[5, 9, 13]^。

多项肿瘤研究表明，前列腺癌^[14, 15]^表达TRPC1、TRPC3、TRPC4和TRPC6蛋白，仅TRPC1和TRPC4参与SOCE，引起肿瘤细胞异常增殖和生长；肝癌^[16]^中可检测到TRPC1和TRPC6，TRPC6介导的SOCE与细胞的恶性增殖有关；TRPC3的表达对卵巢癌^[17]^的发展有促进作用；胃^[18]^及食道癌^[19]^表达TRPC6，乳腺癌^[20]^则表达TRPC3和TRPC6，其表达与细胞增殖有关；而基底细胞癌^[21]^不表达TRPC1和TRPC4，缺乏IP3诱发的SOCE，阻碍肿瘤细胞向正常分化。可见，TRPC1和/或TRPC6与大多数肿瘤关系密切。

关于肺癌组织中TRPC的表达、SOCC构成情况、是否存在SOCE异常以及与肿瘤的关系，目前还未有报道。本研究首先探讨了NSCLC组织中TRPC的表达情况，结果表明，NSCLC组织表达TRPC1、TRPC3、TRPC4和TRPC6 mRNA和蛋白质，并未检测到*TRPC2*、*TRPC5*和*TRPC7*基因的表达。进一步分析发现，TRPC1、TRPC3、TRPC4和TRPC6 mRNA在NSCLC组织中的表达丰度不同，TRPC1和TRPC6高表达，而TRPC3和TRPC4表达丰度远远低于前者。研究^[22]^表明，SOCC是TRPC通道蛋白以同源或异源方式形成的四聚体通道，NSCLC组织中TRPC的这种表达丰度或许同SOCC的组成方式有关，因此推测，NSCLC的SOCC可能主要由TRPC1和/或TRPC6构成，TRPC3^[12]^和TRPC4并不主要参与或不参与构成SOCC。

综上所述，本研究首次发现了NSCLC组织中TRPC的表达谱和相对表达丰度，这些TRPC通道蛋白在NSCLC组织中对癌细胞SOCE的贡献及与肺癌进展的关系还有待进一步研究。
